# RiboTALE: A modular, inducible system for accurate gene expression control

**DOI:** 10.1038/srep10658

**Published:** 2015-05-29

**Authors:** Navneet Rai, Aura Ferreiro, Alexander Neckelmann, Amy Soon, Andrew Yao, Justin Siegel, Marc T. Facciotti, Ilias Tagkopoulos

**Affiliations:** 1 UC Davis Genome Center, University of California-Davis, Davis, CA, USA; 2 Department of Biochemistry & Molecular Medicine, University of California-Davis, Davis, CA, USA; 3 Department of Chemistry, University of California-Davis, Davis, CA, USA; 4 Department of Biomedical Engineering, University of California-Davis, Davis, CA, USA; 5 Department of Computer Science, University of California-Davis, Davis, CA, USA; 6 UC Davis Undergraduate Program, University of California-Davis, Davis, CA, USA

## Abstract

A limiting factor in synthetic gene circuit design is the number of independent control elements that can be combined together in a single system. Here, we present RiboTALEs, a new class of inducible repressors that combine the specificity of TALEs with the ability of riboswitches to recognize exogenous signals and differentially control protein abundance. We demonstrate the capacity of RiboTALEs, constructed through different combinations of TALE proteins and riboswitches, to rapidly and reproducibly control the expression of downstream targets with a dynamic range of 243.7 ± 17.6-fold, which is adequate for many biotechnological applications.

Our ability to reliably forward-engineer novel genetic circuits from libraries of well-characterized genetic parts depends on numerous factors, including but not limited to, the diversity of available parts and knowledge of their activities across variable genetic and functional contexts. Parts encoding regulatory elements (i.e. transcription factors, promoters, ribosome binding sites, terminators, etc.) are particularly important as they typically compose the structural and functional core of a genetic circuit.

Despite recent reports announcing the public release of numerous well-characterized regulatory elements^1^,^2^,^3^ and an associated modular assembly scheme for assembling regulatory modules with predictable output levels comparably, there are still many chemical or environmental signals for which a specific regulatory element is needed. As such, expanding the regulatory toolkit to include flexible, accurate and easy to engineer custom regulators is crucial for both precise control and scaling up synthetic designs. Indeed, most of the synthetic gene circuits have been constructed using a very limited set of orthogonal promoters, namely *P*_Lac_, *P*_Tet_, *P*_BAD_ and *P*_cI_^4^.

Recently, Transcription Activator-Like Effectors (TALEs) proteins and riboswitches have been proved to be versatile tools for the target specific gene regulation^5^,^6^,^7^. TALEs are synthesized and secreted by several species of the plant pathogenic bacteria Xanthomonas^8^ and they are composed of highly conserved tandem repeat domains, each consisting of 33 or 34 amino acids. Individual repeats vary mostly at positions 12 and 13, and amino acids present at these two positions are called repeat variable di-residues (RVDs), each specifying a preference for binding an individual nucleotide^8^,^9^. Repeat domains, each recognizing a specific nucleotide, can be assembled into a specific linear order to target nearly any DNA sequence^10^,^11^ with a length of at least 3 to 4 bp^12^. Riboswitches are non-protein coding regulatory RNAs, typically found in the 5´ untranslated (UTR) region of mRNA, that undergo conformational changes upon binding with ligands to control gene expression at the translational level in a dose dependent manner^13^,^14^. Riboswitches are conceptually comprised of two parts: a ligand specific aptamer domain and an expression platform, which contains a ribosome binding site (RBS) and undergoes structural changes in response to the changes in the aptamer^14^. Ligand-driven conformational changes in the expression platform regulate the translation either by sequestering RBS or by making it accessible^15^. Since riboswitches can be designed with any type of aptamer domain and hence ligand recognition site, their modular nature makes them a powerful tool for the synthetic biology applications^6^,^16^.

Here, we created a new class of control elements, coined RiboTALEs that arise from the fusion of theophylline inducible riboswitches which are well studied and have been widely used to regulate the targeted gene expression^17^,^18^, and TALEs. The RiboTALEs feature multiple input control of virtually any target promoter and they can be tuned to control the basal and maximum expression levels of their targets. To evaluate their utility in bacterial systems, several combinations of RiboTALE repression networks consisting of different RiboTALE and target modules were constructed by fusing different combinations of riboswitches and TALEs in RiboTALE modules, thus targeting different promoters in the constructed target modules. We demonstrate that RiboTALEs can be engineered to regulate the expression of target genes with high specificity and that these hybrid molecules can be used to regulate target promoter activity. In addition, we evaluated the capacity of RiboTALEs to regulate both inducible and constitutive promoter families. Our results argue that RiboTALEs can be an effective and versatile tool for synthetic circuit and genome engineering when a low operational range (up/down activity) can be tolerated, while high specificity and reproducibility is desired.

## Results and discussion

### Design of RiboTALE repression networks

**Figure 1:**
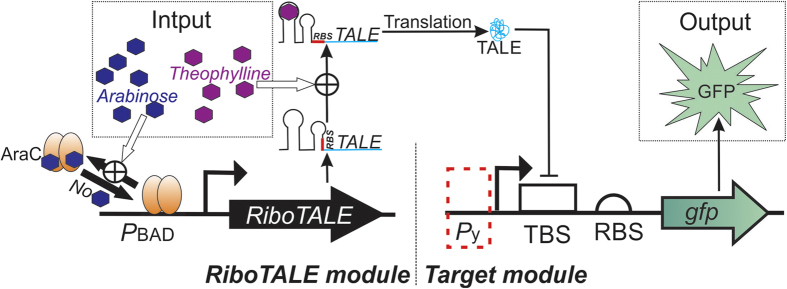
Schematic of a representative RiboTALE repression system. The RiboTALE repression system consists of an inducible RiboTALE module and one or more targets. The RiboTALE module is comprised of a Riboswitch and a *TALE* gene. The RiboTALE module is initially repressed by AraC and is only activated in the presence of arabinose. In the latter case, the RiboTALE expression cassette is expressed, but the mRNA is not translated in the absence of the riboswitch signal (here theophylline). In the presence of theophylline, the riboswitch changes its conformation, exposes the Ribosomal Binding Site (RBS) so that the ribosome can bind, which results in the synthesis of active RiboTALE proteins that in turn bind to the TALE binding site (TBS) present in the target module. *P*_y_ is either *P*_Tet_ or *P*_Const_ promoter.

RiboTALE repression networks were comprised of two modules. The *RiboTALE module* encodes a fusion of a riboswitch and a TALE whose expression is driven from an inducible *P*_BAD_ promoter. The *target module* contains a TALE binding site (TBS) downstream of either a regulated tetracycline (*P*_Tet_) or constitutively (*P*_Const_) expressed promoter (Fig. 1 and Table 1) that drives the expression of a reporter green fluorescent protein (*gfp).* GFP fluorescence was used as a proxy to quantitate the influence of RiboTALE:TBS pairs on the transcriptional output of the target promoter. The four RiboTALE modules, pRibo1TALE1, pRibo1TALE2, pRibo2TALE1, and pRibo2TALE2, were constructed through the combinatorial assembly of two theophylline inducible riboswitches (riboswitch-1 and 2) and genes encoding two TALEs (TALE1 and TALE2) (Table 1). These modules were cloned into the Kan^R^, medium copy number BioBrick plasmid, pSB3K3^19^. Riboswitch-1 (corresponds to riboswitch E in Topp *et al*, 2010 20) and riboswitch-2 (corresponds to clone 8.1* in Lynch & Gallivan, 2009^21^) were previously described to have the same level of maximum fold induction and have 90% DNA sequence similarity. TALE1 and TALE2 were previously described to bind unique 19 bp DNA target sequences (TBS1 and TBS2, respectively) with high (K_D_ = 240 ± 40 nM) and low (K_D_ = 1.3 ± 0.3 nM) dissociation constants, respectively (read TALE1 as I-NIp and TALE2 as II-NNp in Meckler *et al*, 2013)^10^ (Supplementary Table S4 and S5). Two types of *P*_Tet_ based target modules, pTetTBS1 and pTetTBS2, either with TBS1 or TBS2, were constructed. Later, a family of *P*_Const_ based target modules were constructed by using a family of constitutive promoters (parts.igem.org/Promoters/Catalog/Anderson), each with TBS2. (Table 1). Complete RiboTALE repression networks were assembled by co-transforming AraC and TetR producing *E. coli* strain MG1655Z1 with plasmids encoding one each of the RiboTALE and target modules.

Activities of target promoters were measured, by monitoring expression levels of GFP, at different combinations of inducers, arabinose, theophylline, and additional anhydrotetracycline (aTc) in the case of *P*_Tet_ modules. We expect that the activity of the target promoters will depend on the “interplay” between various inducers influencing (a) transcript abundance of RiboTALE, and (b) the translation of the TALE repressor. Level of active TALE can be regulated using arabinose, and theophylline. Arabinose induces *P*_BAD_ promoter, which expresses a fusion of *riboswitch:TALE*. The TALE protein is translated from the transcript of *riboswitch:TALE* only in the presence of riboswitch inducer, theophylline. Apart from these inducers, activity of target promoters will also be governed by the dissociation constant of TALE with its TBS present downstream of the target promoter, and leaky expression of TALEs. We expect that TALE2 will repress its target modules more efficiently than TALE1, due to its lower K_D_. We measured the multi-dimensional control of target promoters, and analyzed how each parameter contributed to the promoter activity^22^.

### Multi-dimensional regulation of inducible *P*_Tet_ target modules

**Figure 2:**
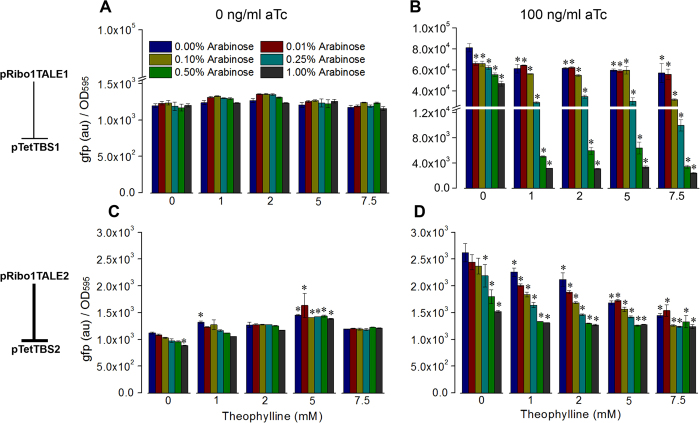
Repression of *P*_Tet_-based target modules. Response of RiboTALE repression system that consists of (**A**) RiboTALE module pRibo1TALE1 and target module pTetTBS1 at 0 ng/ml aTc, (**B**) RiboTALE module pRibo1TALE1 and target module pTetTBS1 at 100 ng/ml aTc, (**C**) RiboTALE module pRibo1TALE2 and target module pTetTBS2 at 0 ng/ml aTc, (**D**) RiboTALE module pRibo1TALE2 and target module pTetTBS2 at 100 ng/ml aTc. K_D_ of TALE1, 240 ± 40 nM; K_D_ of TALE2, 1.3 ± 0.3 nM. Error bars represent standard error of the mean (N = 3). Significant differences between the unrepressed (0% arabinose and 0 mM theophylline) and repressed RiboTALE repression systems are indicated by * (p-value <0.05).

Expression of GFP from *P*_Tet_ containing target modules was regulated by changing the concentrations of three unique inducer molecules (arabinose, theophylline, and aTc). The two control units, *P*_BAD_ and the theophylline-induced riboswitch, were part of the RiboTALE module, while the third unit, *P*_Tet_, was present in the target module. *P*_Tet_ target modules were cloned into the chloramphenicol-resistant high copy number BioBrick plasmid pSB1C3. Two types of *P*_Tet_ target modules were constructed, pTetTBS1 and pTetTBS2. pTetTBS1 is composed of *P*_Tet_:*TBS1*:*gfp* and pTetTBS2 is composed of *P*_Tet_:*TBS2*:*gfp*. pTetTBS1 is recognized and repressed by RiboTALE modules, pRibo1TALE1 and pRibo2TALE1, while pTetTBS2 is recognized and repressed by pRibo1TALE2 and pRibo2TALE2, generating varying levels of repression in the GFP expression. All pairwise combinations of RiboTALE and target modules were co-transformed into *E. coli* MG1655Z1. Responses of target modules were measured at various levels of inputs. *P*_BAD_ was induced at 0, 0.01, 0.10, 0.25, 0.50, 1.0% (w/v) arabinose, riboswitches were induced at 0, 1, 2, 5, 7.5 mM theophylline, and *P*_Tet_ was induced either at 0 or saturated level of aTc (100 ng/ml). In total, for each combination of RiboTALE and target modules, responses were measured at 60 input points. At 0 ng/ml aTc concentration and at various levels of inducers, arabinose and theophylline, reduction in GFP expression was nominal with increasing levels of inducers (Fig. 2A, C and Supplementary Fig. S1A, C). The pRibo1TALE2 and pTetTBS2 pair had 1.3 ± 0.18-fold change in the GFP expression at fully induced pRibo1TALE2 expression and in the absence of aTc (Fig. 2C). This nominal change in the expression is expected as the host strain MG1655Z1 constitutively produces high level of tetracycline repressor TetR, which in the absence of aTc, represses the *P*_Tet_ promoter and consequently narrows the dynamic-response window. At saturating aTc levels (100 ng/ml), different combinations of arabinose and theophylline generated a wide dynamic-response window that spans a 35.1 ± 2.5-fold peak-to-peak activity (Fig. 2B, D and Supplementary Fig. S1D). Cells co-transformed with pRibo1TALE1 and pTetTBS1 demonstrated a highly regulated behavior with a maximum repression of 35.1 ± 2.5-fold at 1% arabinose, 7.5 mM theophylline and 100 ng/ml aTc (Fig. 2B). Cells co-transformed either with pRibo1TALE2 and pTetTBS2 or pRibo2TALE2 and pTetTBS2 also demonstrated appreciable levels of changes in the repression with maximum repression of 2.1 ± 0.14- and 6.3 ± 1.4-folds (Fig. 2D and Supplementary Fig. S1D), respectively. Cells transformed with pRibo2TALE1 and pTetTBS1 did not demonstrate a significant level of change in the response at any concentration of input (Supplementary Fig. S1B). One of the possible reasons for this non-responsiveness may be because of possible mal-functionality of pRibo2TALE1, where fusion of Riboswitch-2 and *TALE1* is unable to form an active TALE1 protein, leading to constitutive expression from pTetTBS1 (Supplementary Fig. S1B).

As shown in the Fig. 2, at 100 ng/ml of aTc, and at comparable levels of arabinose and theophylline, the RiboTALE module pRibo1TALE2 represses its target module more than the pRibo1TALE1 (35.1-fold vs. 2.1-fold, a 16.7-fold increase). Results are in agreement with the measured K_D_s for the respective TALE proteins, with TALE1 having two orders of magnitude higher disassociation constant (240 ± 40 nM) than TALE2 (1.3 ± 0.3 nM) for these recognition sequences. Since TALE2 had higher K_D_ than TALE1, it started repressing respective target modules even in the absence of arabinose and theophylline, and thus generated lower dynamic range than TALE1 (Fig. 2). In all cases except one (binding of pRibo2TALE1 to pTetTBS1, Supplementary Fig. S1B), GFP levels are identical to the basal level when the TALE bind at saturating levels of aTc, arabinose and theophylline.

### Multi-dimensional regulation of constitutive *P*_Const_ target modules

**Figure 3:**
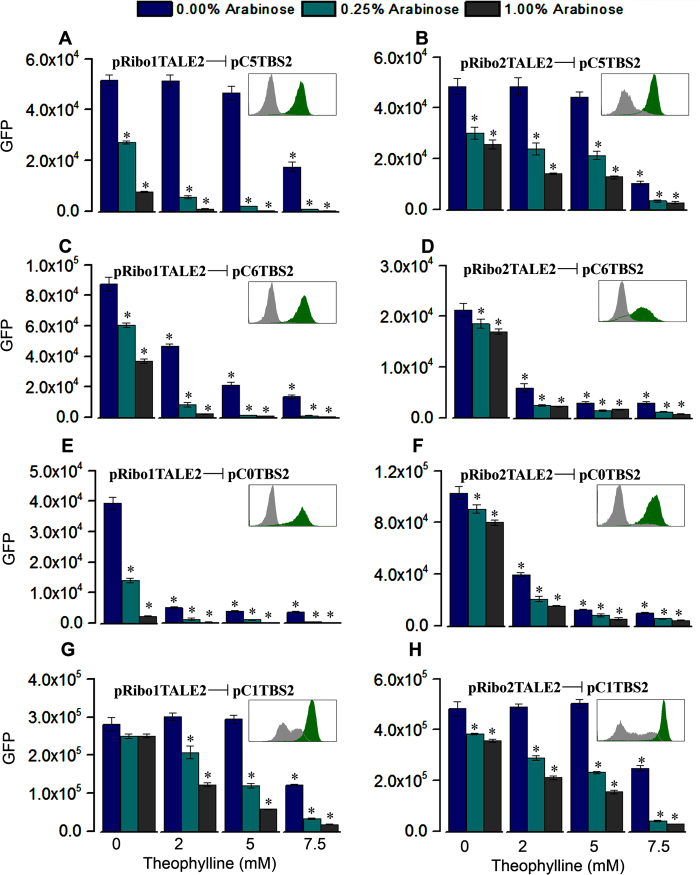
Repression of *P*_Const_-based target modules. Response of RiboTALE repression system that consists of (**A**) RiboTALE module pRibo1TALE2 and target module pC5TBS2, (**B**) RiboTALE module pRibo2TALE2 and target module pC5TBS2, (**C**) RiboTALE module pRibo1TALE2 and target module pC6TBS2, (**D**) RiboTALE module pRibo2TALE2 and target module pC6TBS2. (**E**) RiboTALE module pRibo1TALE2 and target module pC0TBS2, (**F**) RiboTALE module pRibo2TALE2 and target module pC0TBS2, (**G**) RiboTALE module pRibo1TALE2 and target module pC1TBS2, (**H**) RiboTALE module pRibo2TALE2 and target module pC1TBS2. Inset: flow cytometry results; green bar, GFP level at 0% arabinose and 0 mM theophylline; grey bar, GFP level at 1% arabinose and 7.5 mM theophylline; x-axis, GFP level; y-axis, count. Error bars represent standard error of the mean (N = 3). Significant differences between the unrepressed (0% arabinose and 0 mM theophylline) and repressed RiboTALE repression systems are indicated by * (p-value <0.05).

We used a family of Biobrick constitutive promoters (*P*_Const_) to assess the capacity of RiboTALE modules to be used in conjunction with well-known constitutive expression libraries. In total, 4 constitutive promoters were selected based on their expression strengths (Supplementary Table 5). Each of these promoters were targeted by RiboTALE modules composed of TALE2 and either riboswitch-1 or riboswitch-2, resulting in total 8 RiboTALE expression units (Table 1). TBS2 was sandwiched between *P*_Const_ and *gfp.* MG1655Z1 cells co-transformed with a specific combination of RiboTALE and target modules generated different levels of responses, with TALE2-based RiboTALEs achieving more pronounced regulatory effects (Fig. 3). Responses of *P*_Const_ target modules were regulated by changing the concentrations of inducers regulating input points, *P*_BAD_ and riboswitches, of RiboTALE modules. *P*_BAD_ was induced at 0, 0.25, 1.0% arabinose, riboswitches were induced at 0, 2, 5, 7.5 mM theophylline. In total, for each combination of RiboTALE and target modules, responses were measured at 12 input points (Fig. 3). Using different combinations of arabinose and theophylline, we were able to get a peak at 243.7 ± 17.6-fold repression (Fig. 3C). It was also observed that if promoter is stronger, fold repression was smaller. Figure S4 presents the single cell distribution of all 8 *P*_Const_ based RiboTALE expression systems, and it is evident that repression system comprised of pC1TBS2 has maximum expression levels at 0 mM theophylline and 0% arabinose, which is many fold higher than other constitutive promoters. Combinations of pRibo1TALE2 and pC1TBS2, and pRibo2TALE2 and pC1TBS2 generated only 17.2 ± 1.0- and 17.1 ± 0.57-fold change in the expression, respectively (Fig. 3). It was observed that at the highest concentration of inducers (7.5 mM theophylline and 1% arabinose), cells started shifting towards the off-state.

## Effect of TALEs and Riboswitches on the repression dynamics

**Figure 4:**
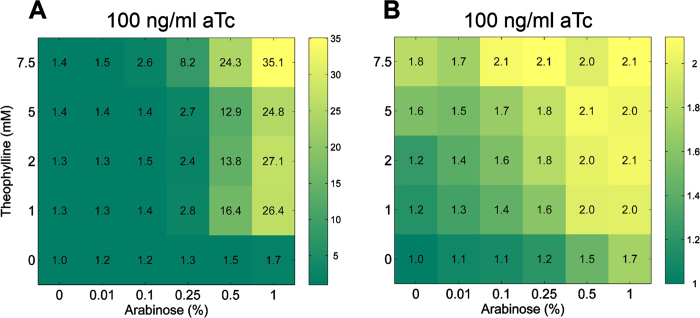
Repression trajectories of *P*_Tet_ based target modules. (**A**) Response of the pRibo1TALE1/pTetTBS1 repression system as a function of arabinose and theophylline concentrations at saturating levels of aTc (100 ng/ml), (**B**) Response of the pRibo1TALE2/pTetTBS2 repression system as a function of arabinose and theophylline concentrations at saturating levels of aTc (100 ng/ml). Color bar indicates fold repression. K_D_ of TALE1, 240 ± 40 nM; K_D_ of TALE2, 1.3 ± 0.3 nM.

**Figure 5:**
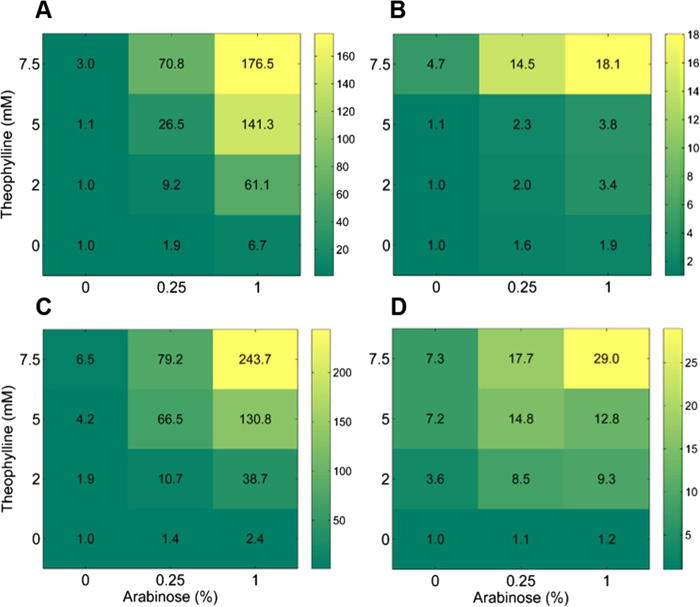
Repression trajectories of *P*_Const_ based target modules. Repression trajectory of RiboTALE repression system that consists of (**A**) RiboTALE module pRibo1TALE2 and target module pC5TBS2, (**B**) RiboTALE module pRibo2TALE2 and target module pC5TBS2, (**C**) RiboTALE module pRibo1TALE2 and target module pC6TBS2, (**D**) RiboTALE module pRibo2TALE2 and target module pC6TBS2. Color bar indicates fold repression.

To provide a comprehensive view of RiboTALE dynamics, we characterized the fold-repression dynamics for each pair of RiboTALE and their target modules. Fold repression was calculated by calculating the ratio of highest GFP fluorescence, which we would expect at lower combinations of arabinose and theophylline concentrations, to that of different arabinose and theophylline concentrations. Figures 4, 5 and S3 represent fold repression generated by different constructs in the explored parameter space. We compared the effect of different TALEs and riboswitches on the repression profile of target promoters, *P*_Tet_ and *P*_Const_. Our results show that different combinations of TALEs and riboswitches produce a unique type of repression trajectory (Fig. 4, 5 and Supplementary Fig. S3). It is also evident from the repression characterization that both TALE1 and TALE2 have similar repression profiles. For instance, repression starts to be pronounced abruptly at higher arabinose (~0.5%) and lower theophylline (~1 mM) concentrations for riboswitch-1 with either TALE1 or TALE2 (Fig. 4A and B). Such pattern is qualitatively common for both the *P*_Tet_ and *P*_Const_ based target modules (Fig. 4A, B and 5A, C). When riboswitch-1 was replaced with riboswitch-2, repression trajectories changed dramatically (compare repression trajectories of riboswitch-1 and 2 derived RiboTALE networks in Fig. 4, 5 and Supplementary Fig. S3). RiboTALE systems employing riboswitch-2 requires higher concentrations of theophylline to fully repress the target promoter than riboswitch-1. Furthermore, RiboTALE repression systems employing riboswitch-1 represses the target module at higher arabinose concentrations even in the absence of the riboswitch inducer (theophylline), while riboswitch-2 based circuits repress target promoter at a higher level of theophylline. Though both riboswitches have 90% homology, riboswitch-1 based RiboTALE modules repress their respective target modules more efficiently.

### Orthogonality of RiboTALE and target modules

**Figure 6:**
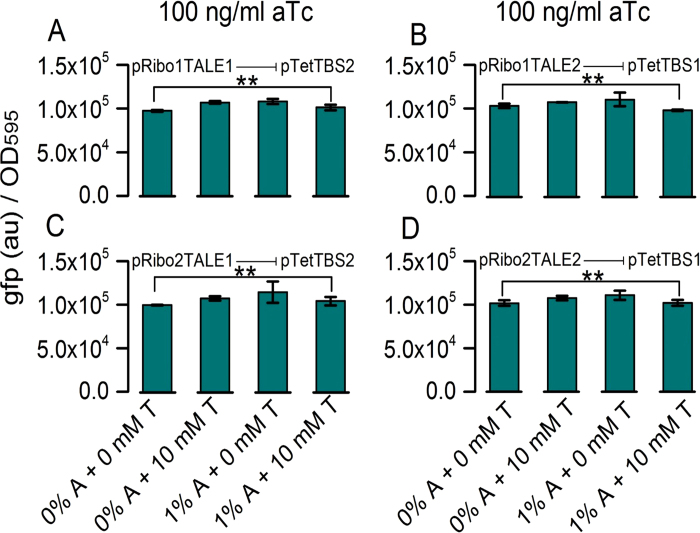
Orthogonality of RiboTALE and target modules. RiboTALE modules do not interfere with off-target modules. Orthogonality of (**A**) pRibo1TALE1 repressing its off-target module pTetTBS2, (**B**) pRibo1TALE2 repressing its off-target module pTetTBS1, (**C**) pRibo2TALE1 repressing its off-target module pTetTBS1, (**D**) pRibo2TALE2 repressing its off-target module pTetTBS1. Error bars represent standard error of the mean (N = 3). All p-values were found to be >0.05 (denoted by ‘**’). A, arabinose; T, Theophylline.

One of the important features of synthetic circuit design is the orthogonality of its genetic parts. We measured the orthogonality of individual RiboTALE and *P*_Tet_ based target modules by co-transforming MG1655Z1 host cells with non-specific pairs of the RiboTALE and target modules (Fig. 6). In these circuits, TALE1 targets TBS2, while TALE2 targets TBS1. In total, the orthogonality of 4 RiboTALE and target module pairs (2 TALEs and 2 riboswitches) were measured at different concentrations of arabinose and theophylline and 100 ng/ml aTc. We found the components to be orthogonal at different induction levels of RiboTALE modules (p-values >0.05 for off-target binding). As expected, the output expression is higher for off-target regulation than on-targeted (Fig. 6 and 2B/D, respectively). This is due to the fact that basal expression in the RiboTALE cognate binding sites present will repress expression from the target modules, even in the absence of the inducers arabinose and theophylline, but not in off-target sites.

## Conclusions

We developed RiboTALEs, a new class of modular and versatile regulatory elements that combine the flexibility and specificity of both TALE and riboswitches to control the expression of any target genes. The addition of riboswitches to TALE control enhance the demonstrated utility of the latter to regulate the expression of almost any target gene^5^,^11^,^23^, by adding an extra point of analog control for modulating the level of repression. Our results demonstrate that repressibility and tunability of designed RiboTALE systems depend on the several parameters including concentrations of inducers, dissociation constants of TALEs, and leakiness of riboswitches. These parameters can be tuned further to generate the desired level of repression of target gene.

Our work is along the same lines and complements efforts that were presented recently to create controllable TALE activators with promising results^24^,^25^. We demonstrate that the resulting libraries can lead to a tightly-controlled and tunable gene expression and with a dynamic range that reached up to 243.7 ± 17.6-fold. The measured operational range which is usually the Achilles’ heel of any riboswitch-based technology, is more surprisingly high for RiboTALEs and doesn’t conform to the notoriety of riboswitches for allowing only a small range of differential control^26^. As our understanding of riboswitches and our ability to engineer aptamer binding domains increases, it will be possible to develop fully orthogonal, highly versatile systems for the control of targeted gene expression^13^. Most importantly, with the addition of each new riboswitch, our arsenal of RiboTALE elements will increase quadratically, as we will be able to mix-and-match with any TALE protein in our library. Adding two or more riboswitches within a single TALE will have an even more profound effect in the number of control elements in our disposal, although this might lead to undesired effects such as non-linearities in the expression dynamics, cross-talk and misfolding of the resulting proteins. Multiplexing of RiboTALEs with other control elements will lead to a much needed powerful arsenal for controlling gene expression and the dynamics of synthetic circuits and will inarguably facilitate the design of more complex and larger circuits, especially if coupled with computer-aided tools and pipelines^27^,^28^,^29^,^30^.

## Materials and methods

### Strains and media

*Escherichia coli* MG1655Z1 (gift from Prof. Shota Atsumi, UC Davis) was used as a chassis for all expression experiments. MG1655Z1 is a derivative of MG1655, and constitutively expresses LacI (~3000 proteins per cell), TetR (~7000 proteins per cell), a spectinomycin resistance selection marker, from the chromosomally integrated Z1 gene cassette, and an AraC from cognate wild type promoter^31^. MG1655Z1 was maintained at −80 °C in LB broth supplemented with 15% glycerol. DH10B or DH5α cells were used for the routine cloning. Strains harboring plasmids were maintained on LB agar plates supplemented with required antibiotic(s). When required, antibiotics chloramphenicol (35 μg/ml), ampicillin (100 μg/ml) and kanamycin (35 μg/ml) were supplemented in media. Co-transformed cells were grown on two antibiotics as both plasmids had different antibiotic resistance markers (Table 1).

### Construction of RiboTALE repression systems

Lists of constructs developed, primers and large oligonucleotide used are reported in Supplementary tables 1, 3 and 4. All constructs were sequenced before performing desired experiments, and later deposited to the BioBrick registry. DNA sequences of all constructs can be downloaded from parts.igem.org using specific BioBrick ID (Supplementary Table S1).

#### Construction of RiboTALE modules

RiboTALE modules were constructed by Golden Gate Assembly^32^. RiboTALE modules were composed of *P*_BAD_ promoter (BioBrick K206000), riboswitch, TALE and a transcription terminator (BioBrick B0015). BioBrick pSB3K3 was used as the plasmid backbone. First, pSB3K3 was made suitable for the Golden Gate assembly, by making it BsaI compatible through site-directed mutagenesis^33^ using primer set pSB3K3_SDM. By site directed mutagenesis, nucleotide ‘G’ of BsaI recognition site was swapped with nucleotide’A’, making it BsaI neutral. This Golden Gate compatible pSB3K3 was deposited to the BioBrick registry and is available as part number K1212002. TALEs were amplified using primer set TALE (Supplementary Table S2). The *P*_BAD_:riboswitch fusions were synthesized with Golden Gate compatible ends as gBlocks gene fragments (Integrated DNA Technologies, Inc) (Supplementary Table S3). Once all DNA fragments were prepared, they were put together by Golden Gate Assembly. For Golden Gate assembly, target DNA sequences were amplified using forward and reverse primers, which added a BsaI restriction site and a 4 bp overhang at both ends of amplified target DNA sequence. The reverse 4 bp overhang was designed to be complimentary to the forward 4 bp overhang of the intended downstream sequence. Likewise, the forward 4 bp overhang was designed to be complimentary to the reverse 4 bp overhang of the intended upstream sequence. Target DNA were amplified using 10 μL of 5X HF Buffer, 1 μL of 10 mM of dNTPs (NEB), 2.5 μL of forward primer, 2.5 μL of reverse primer, 100 ng of template DNA, 0.5 μL DNA Phusion Polymerase (NEB), and ddH_2_O to 50 μl. After Golden Gate PCR amplification with the appropriate primers, amplified parts were gel purified using NucleoSpin Extract II Kit (Macherey-Nagel) and subsequently assembled in a one-pot reaction comprising of 40 fmol of each DNA templates, 0.75 μL BsaI, 0.2 μL 100X BSA, 1 μL T4 DNA ligase (NEB), 2 μL 10X T4 ligase buffer, with ddH_2_0 to 20 μl, and using following programs in the thermal cycler (DYAD, Bio-Rad); (1) 37 °C for 2 min, (2) 16 °C for 3 min, (3) repeat steps 50 times in total, (4) 50 °C for 5 min, (5) 80 °C for 5 min, and (6) 4 °C hold forever. *E. coli* competent cells were transformed with 5-10 μL of Golden Gate products.

#### *Construction of P*_*Tet*_ *target modules*

*P*_Tet_ target modules were comprised of *P*_Tet_ (Biobrick R0040), *TBS*, *gfp*, and a terminator (BioBrick B0015). LVA tagged *gfp* was used to capture the dynamics of network responses. LVA tagged *gfp* was amplified from BioBrick K750000 using primer set GFP_lva, and was made BsaI compatible using site-directed mutagenesis with primer set GFP_lva_SDM (Supplementary Table S2). *P*_Tet_*:TBS* fusions, containing Golden Gate compatible ends, were synthesized as gBlocks gene fragments (Supplementary Table S3). These fragments were cloned in our BsaI compatible vector pSB3K3 (BioBrick K121200), by Golden Gate Assembly as previously described and later assembled *P*_Tet_ target modules were moved into the BioBrick vector pSB1C3.

#### Construction of P_Const_ target modules

*P*_Const_ target modules were comprised of *P*_Const_ (BioBrick J2310x series promoters, x: 0, 1, 5, 6), *TBS*, *gfp_lva*, and a terminator (BioBrick B0015). These modules were made using Standard BioBrick RFC10 Assembly^19^. LVA tagged *gfp* along with TBS were amplified from *P*_Tet_ target modules, using primers XbaI**_**TBS1 and BBa_G00101 to amplify from TBS1 based *P*_Tet_ target module, and primers XbaI**_**TBS2 and BBa_G00101 to amplify from TBS2 based *P*_Tet_ target module (Supplementary Table S2). Final *P*_Const_ target modules were made by replacing RFP of *P*_Const_ (Biobrick J2310x series promoters, x: 0, 1, 5, 6) constructs with these amplified fragments.

### Cell growth profile and fluorescence measurements of *P*_Tet_ based RiboTALE systems

Cells were grown overnight in LB at 37 °C at 150 rpm in an incubator shaker (Innova 44, New Brunswick). Stationary phase cultures were diluted to an optical density measured at 600 nm (OD_600_) ~01 in fresh 0.4% glucose-supplemented M9 salt medium. Then, cells were grown at 37 °C at 150 rpm in an incubator shaker to an OD_600_ ~0.5. Cells were pelleted down and re-suspended in 0.4% glucose-supplemented M9 salt medium. Transparent and flat bottom, 96 well plate (Costar, Corning) was loaded with the 0.4% glucose-supplemented M9 salt medium and required inducers. 40 μL of re-suspended cells were inoculated to reach a final volume of 200 μl. Blank media, and strains without plasmids were used as controls. Cells were grown in Tecan infinite F200 PRO operating at 37 °C and orbital shaking frequency of 244.5 rpm with an amplitude of 2.5 mm. Optical density measured at 595 nm (OD_595_) and GFP were measured every 10 minutes for at least 10 h. GFP was measured using excitation filter 485 ± 20 nm and emission filter 535 ± 25 nm at the gain of 35. All output data has been presented by normalizing GFP fluorescence with the OD_595,_ at 10 h.

### Flow cytometry of *P*_Const_ based RiboTALE systems

*P*_Const_ based RiboTALE expression systems were analyzed using flow cytometer. Cells were grown for 8 h in LB broth at 37 °C and at 150 rpm in an incubator shaker (Innova 44, New Brunswick). 50 μl of grown culture was transferred to 5 ml of 0.4% glucose-supplemented M9 salt medium, and grown overnight in an incubator shaker at 37 °C. Cells were centrifuged and re-suspended in 0.4% glucose-supplemented M9 salt medium. 2 μl of re-suspended cells was transferred to 198 μl of 0.4% glucose-supplemented M9 media enriched with required concentrations of theophylline and arabinose. Cells were grown at 37 °C for 12 h. GFP levels were measured using BD Accuri™ C6 cytometer (BD Biosciences). At least 20,000 events were recorded for each sample. For each event, forward- and side-scatter, as well as GFP levels (488 nm excitation laser; FL1 filter set) were recorded. Cells were selected from a tight forward- and side-scatter gate, and GFP levels were calculated.

### Statistical analysis

All data have been reported as the mean with standard error. Values of the mean were calculated at least from the three separate experiments. P-values were calculated by one-way ANNOVA followed by the Dunnett multiple comparisons test using InStat (GraphPad Software, Inc., USA). A p-value < 0.05 was considered statistically significant.

## Additional Information

**How to cite this article**: Rai, N. *et al*. RiboTALE: A modular, inducible system for accurate gene expression control. *Sci. Rep.* **5**, 10658; doi: 10.1038/srep10658 (2015).

## Supplementary Material

Supplementary Information

## Figures and Tables

**Table 1:** List of plasmids.

Plasmid	Parts	Backbone	Resistance		Source(s)
		***RiboTALE modules***			
**pRibo1TALE1**	*P*_BAD_* + Riboswitch-1 + TALE1	pSB3K3*	Kanamycin		This study,
					Meckler *et al.* 2013
**pRibo2TALE1**	*P*_BAD_* + Riboswitch-2 + TALE1	pSB3K3*	Kanamycin		This study,
					Meckler *et al.* 2013
**pRibo1TALE2**	*P*_BAD_* + Riboswitch-1 + TALE2	pSB3K3*	Kanamycin		This study,
					Meckler *et al.* 2013
**pRibo2TALE2**	*P*_BAD_*Riboswitch-2 + TALE2	pSB3K3*	Kanamycin		This study,
					Meckler *et al.* 2013
				
**pTetTBS1**	*P*_Tet_* + TBS1 + GFP	pSB1C3*	Chloramphenicol		This Study
**pTetTBS2**	*P*_Tet_* + TBS2 + GFP	pSB1C3*	Chloramphenicol		This Study
				
**pC5TBS2**	J23105* + TBS2 + GFP*	pSB1A2*	Ampicillin		This Study
**pC6TBS2**	J23106* + TBS2 + GFP*	pSB1A2*	Ampicillin		This Study
**pC0TBS2**	J23100* + TBS2 + GFP*	pSB1A2*	Ampicillin		This Study
**pC1TBS2**	J23101* + TBS2 + GFP*	pSB1A2*	Ampicillin		This Study

^*^BioBricks ( parts.igem.org).
