# Supplementary material for: RiboTALE: A modular, inducible system for accurate gene expression control
Source: Sci Rep. 2015 May 29;5:10658. doi: 10.1038/srep10658 (PMC4650599; doi:10.1038/srep10658)
Supplement: Supplementary Information [file srep10658-s1.pdf]

RiboTALE: A modular, inducible system for accurate gene expression control

Navneet Rai,[1] Aura Ferreiro,[6] Alexander Neckelmann,[6] Amy Soon,[6] Andrew Yao,[1,4] Justin Siegel,[1,2,3] Marc Facciotti,[1,4] Ilias Tagkopoulos,[1,5*]

[1]UC Davis Genome Center, University of California-Davis, Davis, CA, USA.
[2]Department of Biochemistry & Molecular Medicine, University of California-Davis, Davis, CA, USA.
[3]Department of Chemistry, University of California-Davis, Davis, CA, USA.
[4]Department of Biomedical Engineering, University of California-Davis, Davis, CA, USA.
[5]Department of Computer Science, University of California-Davis, Davis, CA, USA.
[6]UC Davis Undergraduate Program, University of California-Davis, Davis, CA, USA.
*Corresponding author (itagkopoulos@ucdavis.edu).

Supplementary material

Contents

Table S1 Constructs submitted to BioBrick registry 2
Table S2 List of primers 2
Table S3 List of synthesized gBlocks 3
Table S4 Sequences of Riboswitch and TBS 3
Table S5 Strengths of P_{Const} promoters and dissociation constants of TALE proteins 3
Figure S1. Repression of P_{Tet} based target modules. 4
Figure S2. Repression trajectories of P_{Tet} based target modules........ 5
Figure S3. Repression trajectories of P_{Const} based target modules........ 6
Figure S4. Responses of cells expressing RiboTALE modules and P_{Const} based target modules at single cell level........ 7

Table S1 Constructs submitted to BioBrick registry

Construct	BioBrick ID
pRibo1TALE1	K1212014
pRibo2TALE1	K1212011
pRibo1TALE2	K1212015
pRibo2TALE2	K1212012
pTetTBS1	K1212013
pTetTBS2	K1212016
pC0TBS2	K1212021
pC1TBS2	K1212022
pC5TBS2	K1212023
pC6TBS2	K1212024
Golden Gate compatible-<br>-pSB3K3	K1212002

Table S2 List of primers

Primer	Type	Sequence (5' → 3')
TALE	Forward	TTTTTTGGTCTCACAAGATGTCCGACGCTTCGCCGG
TALE	Reverse	AAAAAAGGTCTCAAAGCTCAACCGGTAGGATCCGGA
GFP_lva	Forward	TTTTTTGGTCTCAGACAATGCGTAAAGGAGAAGAACT
GFP_lva	Reverse	GGGCCTTTCTGCGTTTATAGCTTTGAGACCTTTTTT
GFP_lva,_SDM	Forward	CCCAACGAAAAGAGAGATCACATGGTCCTTCTTGAG
GFP_lva,_SDM	Reverse	CTCAAGAAGGACCATGTGATCTCTCTTTTCGTTGGG
XbaI_TBS1	Forward	ATAATGTCTAGATAAACAGATAAATAGACAA
XbaI_TBS2	Forward	ATAATGTCTAGATGAGTGCGGGAGCGTGGGG
BBa_G00101	Reverse	ATTACCGCCTTTGAGTGAGC
pSB3K3_SDM	Forward	CAGTGCTGCAATGATACCGCAAGACCCACGCTCACCGGCTC
pSB3K3_SDM	Reverse	GAGCCGGTGAGCGTGGGTCTTGCGGTATCATTGCAGCACTG

Table S3 List of synthesized gBlocks

Part	Oligonucleotide sequence (5' → 3')
pBAD+<br>Riboswitch-1<br>+GFP	TATAGTGGTCTCAATGCACATTGATTATTTGCACGGCGTCACACTTTGCTAT<br>GCCATAGCAAGATAGTCCATAAGATTAGCGGATCCTACCTGACGCTTTTTAT<br>CGCAACTCTCTACTGTTTCTCCATACCGTTTTTTTGGGCTAGCGGTGATACC<br>AGCATCGTCTTGATGCCCTTGGCAGCACCCTGCTAAGGAGGTAACAACAAGT<br>GAGACCTCGATG
pBAD+<br>Riboswitch-2<br>+GFP	TATAGTGGTCTCAATGCACATTGATTATTTGCACGGCGTCACACTTTGCTAT<br>GCCATAGCAAGATAGTCCATAAGATTAGCGGATCCTACCTGACGCTTTTTAT<br>CGCAACTCTCTACTGTTTCTCCATACCGTTTTTTTGGGCTAGCGGTGATACC<br>AGCATCGTCTTGATGCCCTTGGCAGCACCCCGCTGCAGGACAACAAGTGAGA<br>CCTCGATG
pTet+TBS1+<br>B0034	TTTTTTGGTCTCAATGCTCCCTATCAGTGATAGAGATTGACATCCCTATCAG<br>TGATAGAGATACTGAGCACTAAACAGATAAATAGAAAAGAGGAGAAAGACAT<br>GAGACCAAAAAA
pTet+TBS2+<br>B0034	TTTTTTGGTCTCAATGCTCCCTATCAGTGATAGAGATTGACATCCCTATCAG<br>TGATAGAGATACTGAGCACTGAGTGCGGGAGCGTGGGGAAAGAGGAGAAAGA<br>CATGAGACCAAAAAA

Table S4 Sequences of Riboswitch and TBS

	**Sequences**
**Riboswitch 1**	GGUGAUACCAGCAUCGUCUUGAUGCCCUUGGCAGCACCCUGCUAAGGAGGUAACAACAAG
**Riboswitch 2**	GGUGAUACCAGCAUCGUCUUGAUGCCCUUGGCAGCACCCCGCUGCAGGACAACAAG
**TBS1**	TAAACAGATAAATAGACAA
**TBS2**	TGAGTGCGGGAGCGTGGGG

Table S5 Strengths of P_{Const} promoters and dissociation constants of TALE proteins

**Strengths of P_{Const} promoters***	
**Promoter BioBrick ID**	**Relative promoter strengths***
J23100	1
J23101	0.70
J23105	0.24
J23106	0.47
**Dissociation constants (K_D) of TALEs[1]**	
TALE1	240±40 nM
TALE2	1.3±0.3 nM

*parts.igem.org/Promoters/Catalog/Anderson

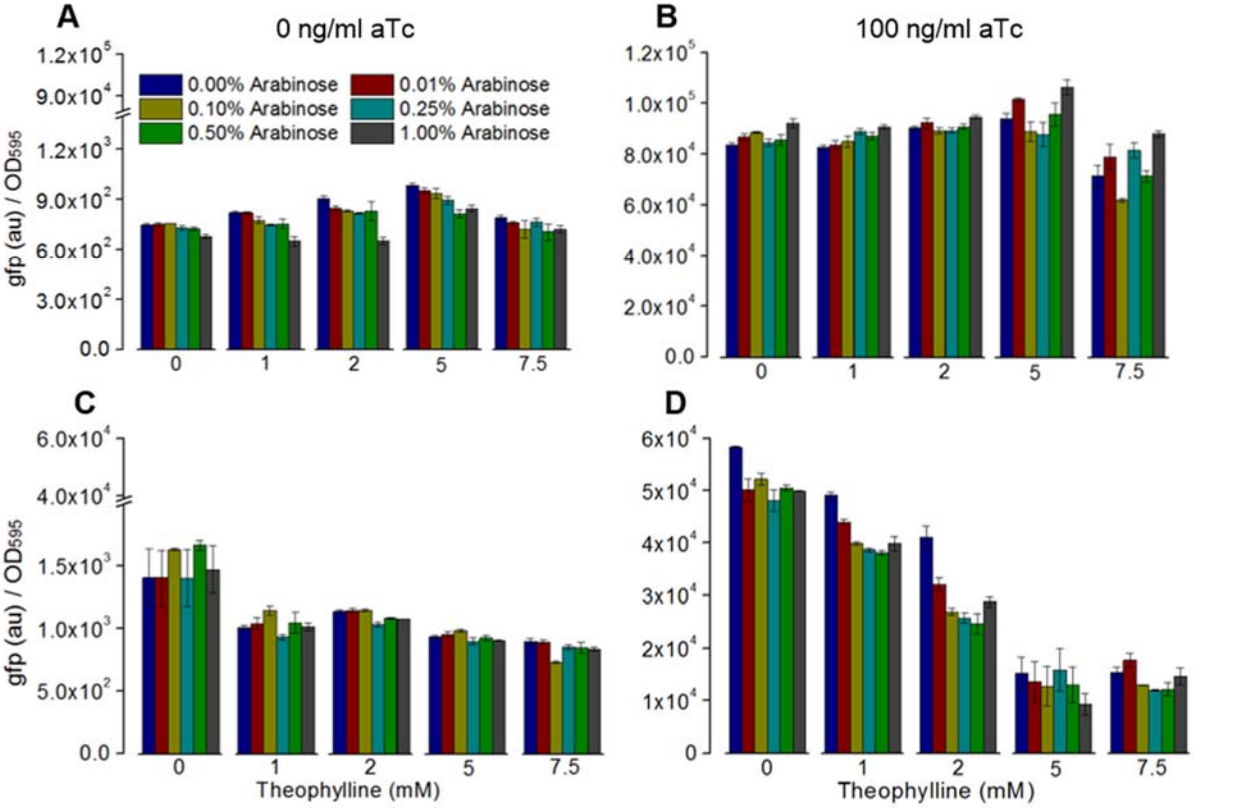

Figure S1. Repression of P_{Tet} based target modules. RiboTALE repression system that consists of (A) RiboTALE module pRibo2TALE1 and target module pTetTBS1 at 0 ng/ml aTc, (B) RiboTALE module pRibo2TALE1 and target module pTetTBS1 at 100 ng/ml aTc, (C) RiboTALE module pRibo2TALE2 and target module pTetTBS2 at 0 ng/ml aTc, (D) RiboTALE module pRibo2TALE2 and target module pTetTBS2 at 100 ng/ml aTc. K_D of TALE1, 240±40 nM; K_D of TALE2, 1.3±0.3 nM. . Error bars represent standard error of the mean (N=3).

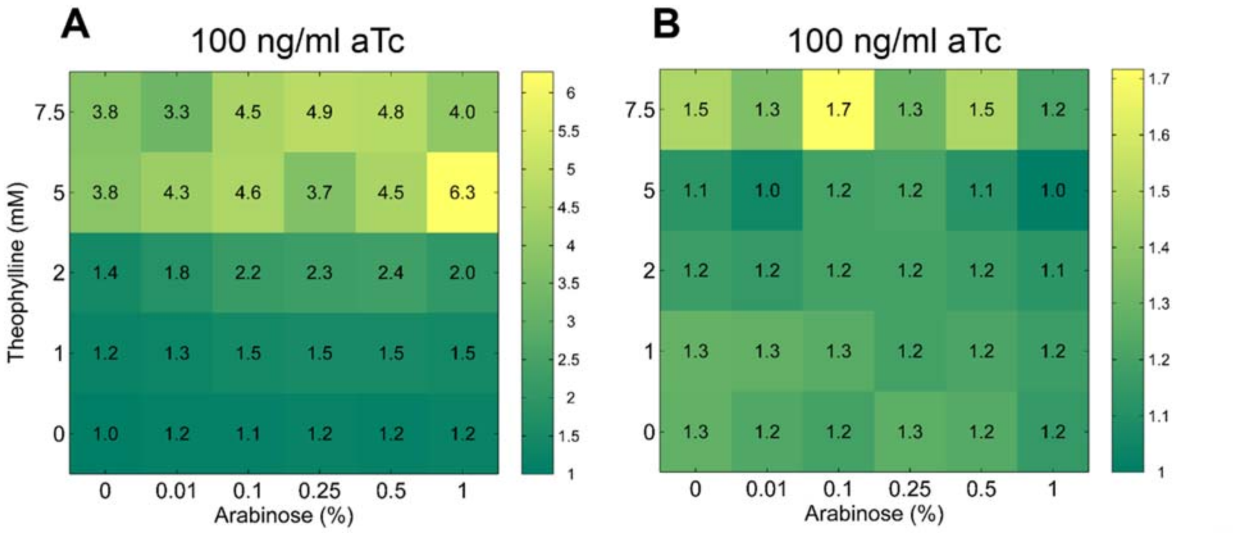

Figure S2. Repression trajectories of P_{Tet} based target modules. Repression trajectory, at saturating levels of aTc (100 ng/ml), of RiboTALE repression system that consists of (A) RiboTALE module pRibo2TALE1 and target module pTetTBS1, (B) RiboTALE module pRibo2TALE2 and target module pTetTBS2. Color bar indicates fold repression. K_D of TALE1, 240±40 nM; K_D of TALE2, 1.3±0.3 nM.

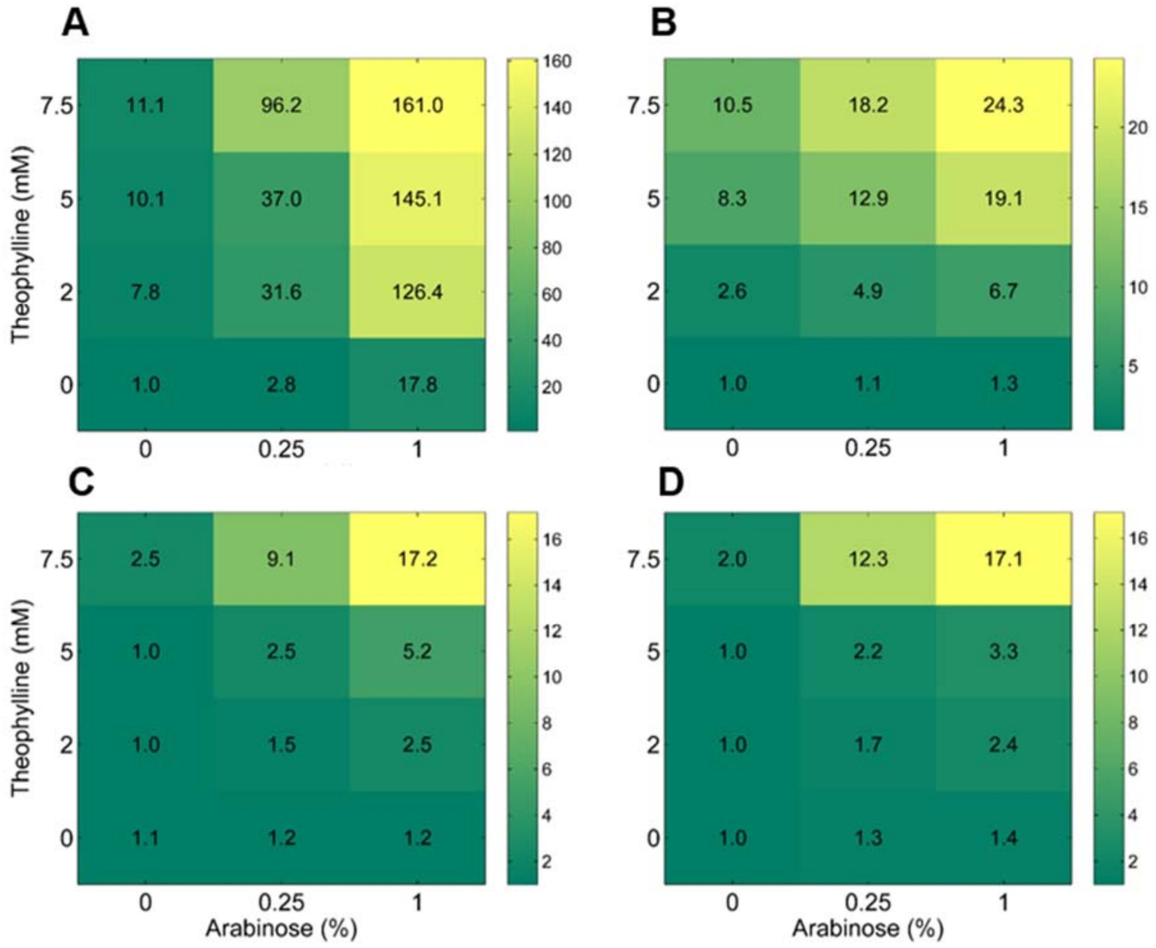

Figure S3. Repression trajectories of P_{Const} based target modules. Repression trajectory of RiboTALE repression system that consists of (A) RiboTALE module pRibo1TALE2 and target module pC0TBS2, (B) RiboTALE module pRibo2TALE2 and target module pC0TBS2, (C) RiboTALE module pRibo1TALE2 and target module pC1TBS2, (D) RiboTALE module pRibo2TALE2 and target module pC1TBS2. Color bar indicates fold repression.

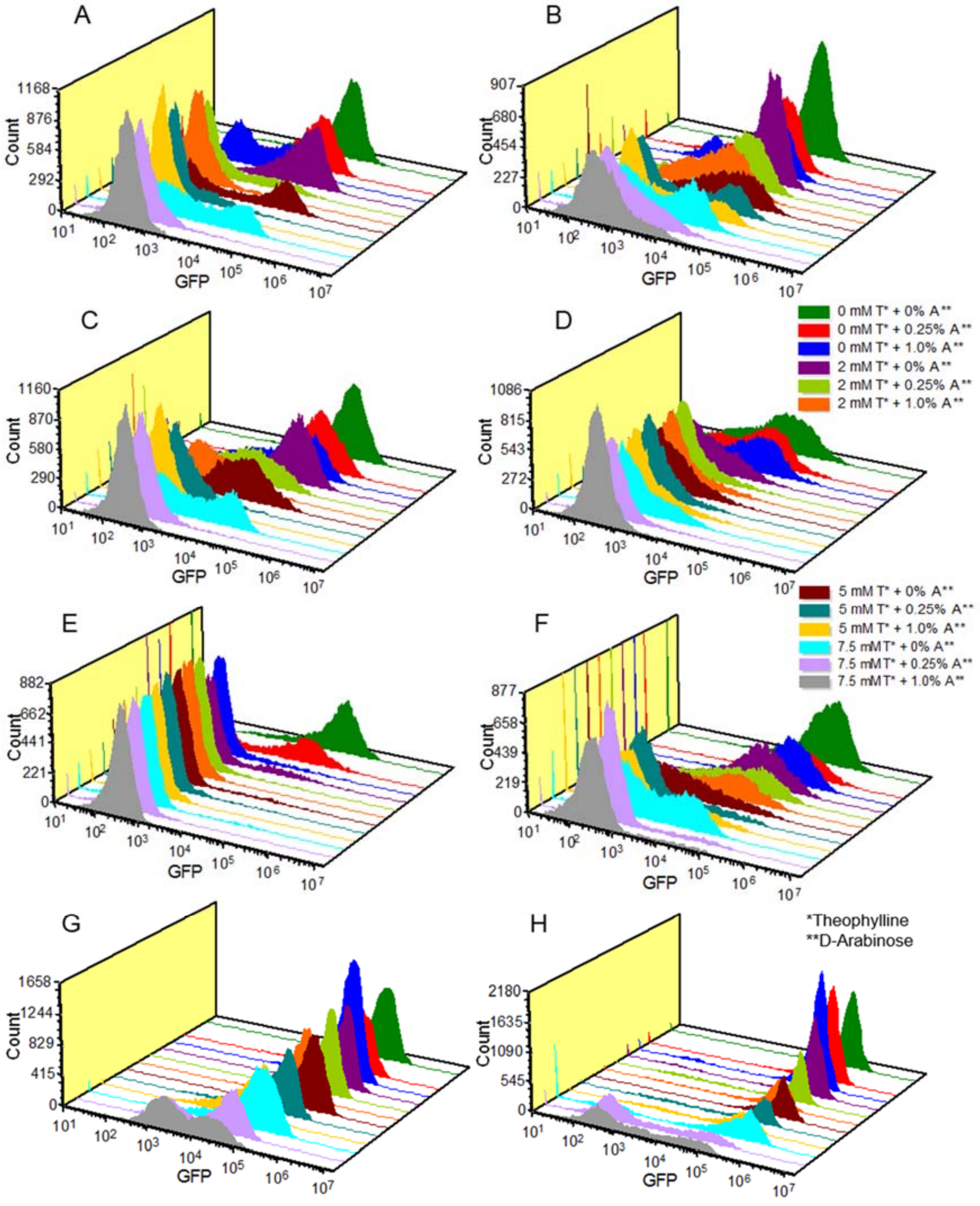

Figure S4. Responses of cells expressing RiboTALE modules and P_{Const} based target modules at single cell level. (A) pRibo1TALE2 and pC5TBS2, (B) pRibo2TALE2 and pC5TBS2, (C) pRibo1TALE2 and pC6TBS2, (D) pRibo2TALE2 and pC6TBS2. (E) pRibo1TALE2 and pC0TBS2, (F) pRibo2TALE2 and pC0TBS2, (G) pRibo1TALE2 and pC1TBS2, (H) pRibo2TALE2 and pC1TBS2.

References

1 Meckler, J. F. *et al.* Quantitative analysis of TALE-DNA interactions suggests polarity effects. *Nucleic Acids Res* **41**, 4118-4128 (2013).
